# Efficiency of Differently Processed Membranes Based on Cellulose as Cationic Dye Adsorbents

**DOI:** 10.3390/nano10040642

**Published:** 2020-03-30

**Authors:** Laura Maleš, Darinka Fakin, Matej Bračič, Selestina Gorgieva

**Affiliations:** 1 Institute of Engineering Materials and Design, Faculty of Mechanical Engineering, University of Maribor, Smetanova ul. 17, 2000 Maribor, Slovenia; laura.males@student.um.si (L.M.); darinka.fakin@um.si (D.F.); matej.bracic@um.si (M.B.); 2 Institute of Automation, Faculty of Electrical Engineering and Computer Science, University of Maribor, Koroška cesta 46, 2000 Maribor, Slovenia

**Keywords:** cellulose membrane, cellulose nanofibrils, bacterial cellulose, adsorption efficiency, cationic dyes

## Abstract

In order to minimize the pollution caused by the reuse of textile dyes, technologies and materials have been developed that purify waste water in an efficient and cost-effective manner before it is discharged into a water body. In this context, the presented research investigates the potential of two types of fully cellulose-based membranes as adsorbents for cationic dyes used in the textile industry. The first type combines cellulose nanofibrils (CNFs) and carboxymethylated cellulose (CMC) using the solvent casting process and an esterification coupling reaction, while the second type uses commercial bacterial cellulose (BC) in a native and sodium periodate-treated form (BCox). The corresponding membranes were comprehensively evaluated by means of Fourier Transform Infrared (FTIR) Spectroscopy. Results confirm the esterification process within the CNF/CMC membranes, as well as BC oxidation after periodate treatment, as shown by bands at 1726.2 cm^−1^ and 895 cm^−1^, respectively. The Potentiometric Titration shows the highest total negative charge of 1.07 mmol/g for 4CNF/4CMC, which is assigned to the presence of COO^−^ within CMC polymers, and lowest (0.21 mmol/g) for BCox. The Contact Angle Goniometry data confirm the hydrophilicity of all membranes, and the angle increased from 0 ° (in pure BC) to 34.5 ° in CMC-rich and to 31.4 ° in BCox membranes due to the presence of CH_2_COO^−^ and CHO groups, respectively. Confocal Fluorescent Microscopy (CFM) demonstrated the highest µ-roughness in 4CNF/4CMC, while Scanning Electron Microscopy (SEM) depicted diverse morphological features between the membranes, from ultrafine nanofiber networks (in BC and BCox) to larger fiber bundles connected within the polymer phase in CNF/CMC membranes. The adsorption experiment followed by UV–VIS spectroscopy, showed ~100% dye removal efficiency in both CNF/CMC-based membranes, while BC and BCox adsorbed only 24.3% and 23.6%, respectively, when anthraquinone dye was used. Azo dye was only adsorbed with an efficiency of 7–9% on CMC/CNF-based membranes, compared with 5.57% on BC and 7.33% on BCox membranes. The adsorption efficiency at equilibrium was highest for BC (1228 mg/g) and lowest for 7CNF/1CMC (419.24 mg/g) during anthraquinone dye adsorption. In the case of azo dye, the BCox was most effective, with 445.7 mg/g. Applicability of a pseudo second-order model was confirmed for both dyes and all membranes, except for BCox in combination with azo dye, showing the fastest adsorption rate in the case of the 7CNF/1CMC membrane.

## 1. Introduction

Dyestuff waste water is an unacceptable effluent discharged from the textile, paper, leather, printing, and other industries, which is largely difficult to break down due to the chemical stability of dyestuffs. It is estimated that up to 15% of the dyes are left unused after the dyeing process of textiles and are released as such, posing a high toxic risk to aquatic animals and humans [1]. With the desire for a sustainable and ecologically oriented society, the re-use of dyes will significantly minimise this type of pollution, which is being developed for various technologies to treat these persistent waste waters in an efficient and cost-effective way before they are discharged into a watercourse. Various methods for removing dyes include membrane separation (ultrafiltration, reverse osmosis) [2], flocculation [3], electrocoagulation [4], precipitation [5], bio sorption [5], catalytic oxidation [6], ozonation [7] and adsorption [8]. The latter is one of the simplest in terms of efficiency, versatility (a wide range of pollutants can be adsorbed), and simplicity of design [9,10,11].

Membrane adsorption provides a versatile platform for the capture of dye molecules. The membranes can be composed of various organic and inorganic components and mixtures thereof, including low-cost biomass and solid wastes from agriculture and industry (bread nut shells, rice husks and straw, willow branches, peanut shells, bagasse, orange peel), clay minerals (e.g., montmorillonite) and zeolites, synthetic polymers (e.g., nylon 6.6), chitosan, manganese oxide/ polyvinyl chloride mixtures, etc. Due to the demand for environmentally friendly materials, the use of cellulose as the most common biopolymer is of great importance. Known cellulose-based nanomaterials, i.e., cellulose nanocrystals (CNCs) [12] and cellulose nanofibrils CNFs [13], are proven adsorbents with good capacity for cationic pollutants (dyes, heavy metal ions), both of which are top-down processed from wood pulp by chemical and/or mechanical means. Natural cellulosic fibers are even weakly anionic (the acidic groups originate from cell wall constituents [14]), and are commonly unable to take up cationic dyes to a significant extent since cationic dyes are neither planar nor sufficiently large for sufficient substantivity and affinity, which is a disadvantage when the dying process is considered. Similarly, their adsorption on cellulose-based adsorbents is rather limited. However, due to the reactivity of abundant OH groups, the ability of cellulose-based adsorbents to adsorb cationic dyes can be increased by suitable modification with functional groups of higher affinity, mainly those rich in nitrogen, sulphur, and oxygen [15]. This may enhance the adsorption capacities by an order of magnitude [16]. Moreover, the CNFs have a film-forming capacity as a result of the interfibrillar hydrogen bonding network formed after drying of CNF dispersions [17]. However, the drying process results in non-uniformly distributed, aggregated CNFs within processed films, forming weak places, which disintegrate in water. This can be overcome by combination with anionic polymers such as carboxymethylated cellulose (CMC). This cellulose derivative has excellent compatibility with water and can form hydrogels, is resistant to high temperatures and pH 2–11, is biodegradable, and offers good adsorption capacities. As an anionic polysaccharide, which contains abundant carboxyl groups, it can easily adsorb both cationic dyes and heavy metals. CMCs have been used for dye adsorption when combined with acrylic acid by graft polymerization [18], with montmorillonite [19], and metal–organic framework beads for metal removal [20]. Bacterial cellulose (BC) is another pure, cellulose-based, ultra-fine, nanofibrillar material with an exclusive combination of properties such as high crystallinity (84–89%) and a high degree of polymerization, a high surface area (high aspect ratio of fibers with a diameter of 20–100 nm), high flexibility and tensile strength (elastic modulus of 15–18 GPa), high water-holding capacity (more than 100 times its own weight), etc. This biopolymer is mainly produced by the species *Komagataeibacter xylinus* [21]. BC itself has been investigated as an adsorbent for the anionic dye acid fuchsine [22], or even used in the processing of activated carbon for the removal of methylene blue with a maximum capacity of 505.8 mg/g [23]. 

The aim of the present study was to compare the efficiency of different membranes based on cellulose (cellulose derivative CMC, as well as nanofibrillated CNFs and BC) as adsorbents of anthraquinone (C.I. Basic Blue 47) and azo (C.I. Basic Yellow 29) cationic dyes. The azo and anthraquinone dyes in general constitute the largest and second largest group of colorants, respectively, used in industry [24]. For this purpose, two types of cellulose-based membranes were processed: the first using a combination of CNF and CMC, and the second using commercial BC and its oxidised form by means of sodium periodate. The physic–chemical and morphological aspects of the membranes were evaluated by Attenuated Total Reflectance–Fourier Transform Infrared (ATR–FTIR) Spectroscopy, Contact Angle Goniometry, Potentiometric Titration, Confocal Fluorescence Microscopy (CFM), and Scanning Electron Microscopy (SEM). UV–VIS Spectroscopy was used to determine the adsorption efficiency and kinetics of the two types of cationic dyes. 

## 2. Experimental Section

### 2.1. Materials

**Figure 1:**
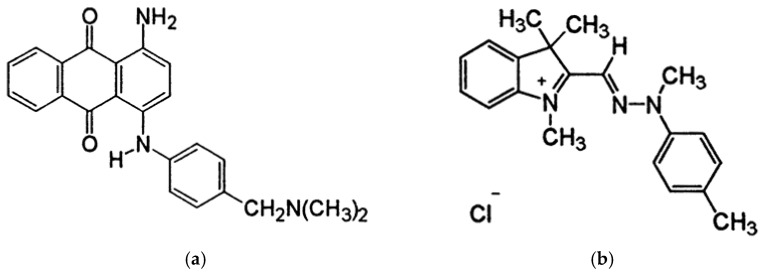
Chemical structure of (**a**) anthraquinone dye C.I. Basic Blue 47 and (**b**) azo dye C.I. Basic Yellow 29.

The CNFs were supplied by the University of Maine (Orono, ME, USA). They are produced from bleached birch pulp, in the form of 3.0 wt.% aqueous gel. The nominal fiber width is 20–50 nm, and lengths reach several hundred microns. CNFs specific surface area as measured by BET is 31–33 m^2^/g, while the density is 1.0 g/cm^3^. The CMC with *M*w=250 000 g/mol and degree of substitution (DS) of 0.65–0.85, anhydrous citric acid (CA), and sodium periodate (NaIO_4_) were purchased from Sigma Aldrich (St. Louis, Missouri, United States) and used without further purification. Purified and autoclaved BC membranes were obtained from Fzmb GmbH (Research Center of Medical Technology and Biotechnology, Bad Langensalza, Germany). BC is produced by the gram-negative bacterium *Gluconacetobacter xylinum*. Nanofibrils mainly consist of cellulose type I, and the overall crystallinity index (including crystalline and amorphous segments) is 0.83 [25]. The diameter of BC nanofibrils is in the 30–60 nm range (as obtained by high-resolution SEM images). Two cationic dyes, C.I. Basic Blue anthraquinone dye 47 *(M*w of 371.43 g/mol) (Figure 1a) and C.I. Basic Yellow azo dye 29 (*M*w of 341.88 g/mol) (Figure 1b), were purchased from the American Association of Textile Chemists and Colorists (NewYork, NY, USA).

### 2.2. Preparation of Membranes 

The CNF/CMC membranes were processed on the basis of a method already described in the literature [8]. In brief, a 1% *w*/*v* CMC solution was prepared in a 24 h dissolution process under moderate stirring. In a separate glass beaker, 2% *w*/*v* CNF dispersion was prepared with a homogeniser (< 5 min at 1000 min^−1^) in order to avoid the presence of large aggregated CNF bundles. Shortly before mixing the two components, the 5% *w/w* CA water solution was prepared, the concentration calculated according to the dry mass of the CNFs and CMC in the final volume for the casting (i.e., 8 mL). Two separate mixtures with the designations 4CNF/4CMC and 7CNF/1CMC were prepared, in which the mass ratios of the two component stock solution (CMC) and suspension (CNF) were listed. For better mixing, 4 mL distilled water and 1.5 mL CA solution were added to the two mixtures. The suspensions were poured on glass Petri dishes with *d* = 80 mm and dried at 60 °C for 24 h and additionally for 2 h at 100 °C.

For the preparation of oxidised BC membrane (further denominated as BCox), square pieces of membrane with dimensions 40 × 40 × 0.17 mm^3^ were immersed in glass beakers with 10 mL 0.5% *w*/*v* NaIO_4_ prepared in deionized water immediately before use. The beakers were covered with aluminium foil and placed on a magnetic stirrer for 24 h. To complete the reaction, the membranes were thoroughly rinsed with deionized water and partially dried at 60 °C (the membranes were not completely dry).

### 2.3. Characterization of Membranes

#### 2.3.1. Attenuated Total Reflectance–Fourier Transform Infrared (ATR–FTIR) Spectroscopy

ATR–FTIR was performed on an IR Perkin–Elmer spectrophotometer with a Golden Gate ATR (Waltham, MA, USA) attached to a diamond crystal. The spectra were collected at ambient conditions from the accumulation of 16 scans with a resolution of 4 cm^−1^ over a range of 4000–650 cm^−1^, with the air spectrum subtraction performed in parallel as a background. The Spectrum 5.0.2 software program (Waltham, MA, USA) was used for data analysis. The spectral data were treated by baseline correction followed by normalization to the “internal standard” peak at ~2890 cm^−1^ (C–H stretching in CH_2_) to account for confounding factors such as varying thicknesses of samples.

#### 2.3.2. Potentiometric Titration

pH-potentiometric titration of CNF water dispersions, being the same for the selected membranes, was performed for quantifying the processing-dependent surface charge contribution. The titration was carried out using a dual-burette automatic titrator T70 (Mettler Toledo, Zurich, Switzerland) filled with 0.1 M HCl and 0.1 M KOH titrants. All the solutions were prepared in deionized water. The titration was performed in a forth (acidic to alkaline) and back (alkaline to acidic) manner, between 2.5 < pH < 11 in an inert (N_2_) atmosphere. The ionic strength was kept constant (0.1 M) by the addition of 3 M KCl. Different titrant volumes (0.001–0.25 mL) were dynamically added within 30- to 180-s periods. A blank HCl–KOH titration was carried out under the same conditions. The pH value was measured using a Mettler Toledo InLab Reach (Zurich, Switzerland) combined glass electrode. Details on surface charge calculations can be found elsewhere [26].

#### 2.3.3. Contact Angle Goniometry 

To assess the hydrophilicity of membranes, the water contact angle was measured with an OCA15+ goniometer system (Dataphysics, Filderstadt, Germany) using SCA 20 program software (Dataphysics, Filderstadt, Germany) and the sessile drop method. All measurements were performed with Milli-Q water (Merck Millipore, Burlington, MA, USA) at ambient temperature with a drop volume of 3 μL. Images were captured immediately after the water drop reached the solid surface of the membrane. The specified angle value measured in same time frame as image capturing was the average of at least five drops of liquid per surface. Each film was measured at three positions, from which the mean value and standard deviations were calculated.

#### 2.3.4. Confocal Fluorescent Microscopy (CFM) Imaging

Visualization of the membranes in the wet state was performed by CFM imaging using cellulose autofluorescence. For this purpose, the fully wet membranes (previously immersed in water until maximum swelling) were positioned on a transparent glass holder above the 20 x objective of a Leica TCS SP5 II inverter CFM unit (Wetzlar, Germany). The wavelengths of excitation (λ_ex_ = 419 nm) and emission (*λ_em_* ~512 nm) were used to obtain the fluorescent signal. High-resolution images (1024 × 1024 pixels) were achieved by light amplification adjustment and 8-fold line averaging. Each membrane was depicted at several positions in order to check the uniformity. Transmitted light images were captured in parallel using a Dodt detector (Leica, Wetzlar, Germany) with the same excitation laser as for the fluorescence images. The images from the fluorescence channel were analysed by the ImageJ program plug-ins: Interactive 3D Surface Plot and Surf-CharJ q1 and for topographic roughness presentation and quantitative characterization, respectively. 

#### 2.3.5. Scanning Electron Microscopy (SEM) Imaging

The morphology of the different cellulose membranes was evaluated by SEM imaging on a Supra 35 VP microscope (Carl Zeiss, Yena, Germany) using secondary electron mode and up to 150 K magnification. Before imaging, all membranes were sputtered with a thin layer of palladium.

#### 2.3.6. UV–VIS Spectroscopy

The adsorption parameters, adsorption amount (Qt), and percentage of dye removal of cellulose-based membranes against two cationic dyes were determined in a batch equilibrium experiment. The dry weight of the membranes was measured before immersion in 100 mL of one of the dye solutions with a concentration of 5 mg/L and pH adjusted to 4 using 0.1 M NaOH and 0.1 M HCl. The adsorption was kept under isothermal conditions, at 23 °C and continuous shaking of 20 min^−1^. After specified incubation intervals, i.e., after 3, 5, 10, 30, 60, 90, 120, 180 min, and 24 h, the absorbance at 580 nm (for C.I. Basic Blue 47) and at 431 nm (for C.I. Basic Yellow 29) was measured with a Cary UV–VIS spectrophotometer (Agilent, Santa Clara, CA, USA). The removal of dye per unit of membrane dry weight (mg/g) and as a percentage (%) was calculated using Equations (1) and (2), respectively:

(1) Qt=(C0−Ct) V/m(g/kg)

(2) $$Removal=\frac{\left(A_{0}-A_{t}\right)}{A_{0}}\times 100\left(\%\right)$$

where *A*_0_ and *A_t_* are the absorbance’s of the initial dye solution and the solutions after the respective immersion times *t* (min), respectively, *V* is the volume of the dye solution (mL), and *m* is the mass of the membrane (g). The amount of dye adsorbed after a certain time (*Qt*) was plotted against time. The pseudo-first and pseudo-second order kinetic models were used to calculate the kinetic parameters, i.e., the kinetic constant *k* and the theoretical equilibrium adsorption amount *Qt* (*th*), and the *R*^2^ value was used to determine the goodness-of-fit with the linear regression model.

## 3. Results and Discussion

### 3.1. Esterification and Oxidation within Cellulose-Based Membranes

**Scheme 1:**
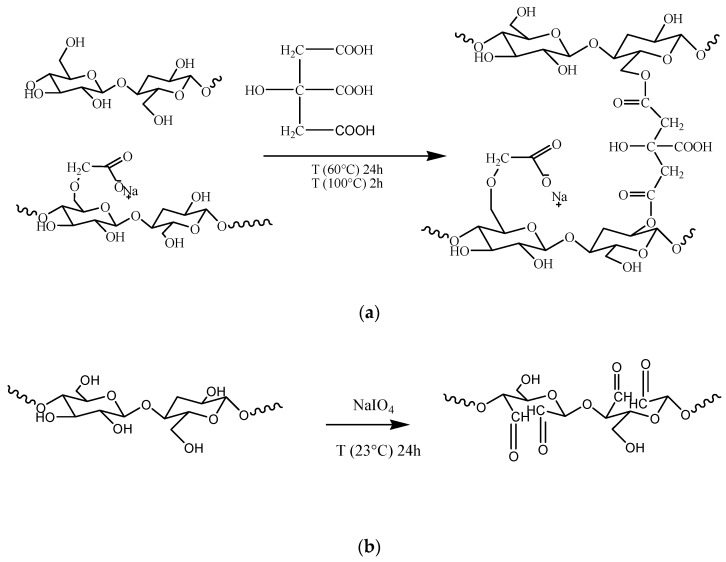
(**a**) Tentative mechanism for CA-mediated CMC coupling with CNF and (**b**) sodium periodate oxidation of BC.

At a particular concentration, the CNF dispersions can be easily converted into self-standing, flexible films [27]; however, they lack wet stability and homogenicity due to the presence of irregular aggregates related to high-length heterogenicity coming from top-down synthesis. The incorporation of the hydrophilic polymer CMC into the CNF dispersion greatly facilitated the dispersibility of nanofibrils as will be demonstrated later by SEM micrographs, and at same time CMC, acted as a filler due to the compatibility and similarities in surface energies of both components [28]. However, the solvent casted membranes from CNF/CMC suspensions failed after aqueous exposure due to the lack of chemical integration and extremely high swelling, which led to a weakening of the interfibrillar bonds [29]. Tricarboxylic acid (citric acid, CA) was used as an esterification agent, which, according to literature [30,31], can support CMC coupling to CNF via available hydroxyl groups, as shown in simplified Scheme 1a. The CA may react with each of the three hydroxyl groups in the anhydroglucose monomers, preferably with those at the C-6 position [32], as well as with the reducing ends of the carbohydrates, resulting in mono-, di-, and tri-esters [33]. 

The periodate-mediated oxidation of BC proceeded through redox cleavage of vicinal (C2 and C3) glycols, which yielded a product with aldehyde groups in positions C2 and C3 of the glucopiranose unit, i.e., 2,3-dialdehyde BC [25], as presented in Scheme 1b. This reaction is thought to proceed via a cyclic di-ester of periodate ion with vicinal hydroxyls, which subsequently undergoes an intramolecular redox process with C–C bond cleavage of the cellulose glucose units.

**Figure 2:**
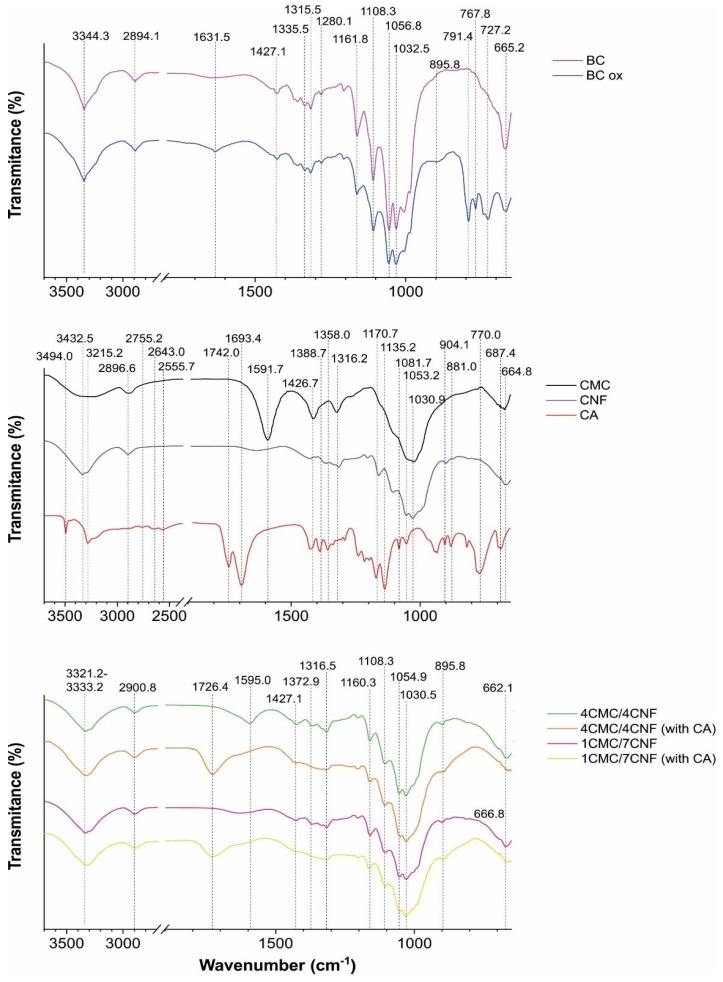
ATR–FTIR spectral lines of components (CMC, CNF, and CA) and cellulose membranes. BCox is the designation of the BC membrane after NaIO_4_ treatment. The CNF/CMC membranes with different mass ratios of components (4/4 and 7/1) and the addition of 5% CA are accordingly designated.

The ATR–FTIR data of components used in CNF/CMC membranes casting (Figure 2a) revealed typical bands related to their components. The broad signal at 3200–3600 cm^−1^ is related to O–H vibrations in CNF and CMC, ~2890 cm^−1^ to C–H stretching in CH_2_, 1742 cm^−1^ and 1693.4 cm^−1^ in anhydrous CA are assigned to ester and carboxylate stretch vibration, respectively, and the bands in the 1000–1070 cm^−1^ region identify primary (~1030 cm^−1^) and secondary (~1055 cm^−1^) alcohols in CNF and CMC. Two vibrations at 1595 cm^−1^ and 1427 cm^−1^, related to asymmetric and symmetric vibration of COO^−^ in CMC, were present in the 4CNF/4CMC membrane before CA addition. The CA-mediated esterification was evident by a new band at 1726.2 cm^−1^ in both CNF/CMC membranes (4/4 and 7/1) prepared with 5 wt.% of CA being assigned to the C=O ester band [34]. This vibration was missing in the CMC, CNF, and CA references and in CA-free membranes. Indeed, the wet, acidic pH (~2) conditions during processing were found to favor the esterification process [35], which in our case was identified after 24 h exposure at 60 °C, with a subsequent 2 h at 100 °C. 

The coupling process also shifted the position of the COO^−^ vibration related to CMC (in the 4CNF/4CMC membrane), from 1595 cm^−1^ towards a higher wavenumber, where the new position partially overlapped with the ester bond at 1726.4 cm^−1^. This is mainly related to increased acidity, which causes protonation of COOH. Small changes in OH vibration in the 3321.2 cm^−1^ – 3333.2 cm^−1^ region are also evident as peak broadening due to high overlap of component-related vibrations. The periodate-treated BC exhibited new bands in the fingerprint region, particularly at 895.8 cm^−1^, related to the aldehyde group and its hemiacetal/hydrated form, as periodate oxidation may involve the formation of free or hydrated aldehydes, hemialdols, and hemiacetals [36] with simultaneous hydrolytic cleavage of glycoside bonds affecting BC crystallinity. We assume that the absence of the characteristic carbonyl (C=O) band at ~1700 cm^−1^ in the oxidised BC membrane was a consequence of a low oxidation degree due to the relatively low periodate concentration used in order to not significantly affect the crystallinity. Herein, the total crystalline index (TCI) was calculated from the ratio of the 1360 cm^−1^/2894 cm^−1^ bands area, which was 0.83 for non-treated BC, while 0.5% periodate oxidation reduced it to 0.74. Other indicators of crystallinity change were bands at ~1427 cm^−1^ and ~895 cm^−1^, which were directly related to crystalline and amorphous structures, respectively, again demonstrating an increase in the amorphous part at the expense of crystallinity after periodate oxidation.

### 3.2. Charge, Hydrophilicity, and Morphology of Membranes

**Figure 3:**
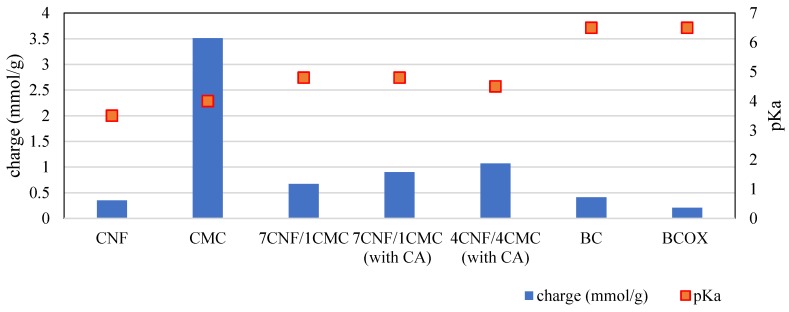
Potentiometric titration data of components (CMC, CNF) and cellulose membranes.

As can be seen from Figure 3, CMC (3.51 ± 0.17 mmol/g) exhibited a 10-fold higher charge than CNF (0.35 ± 0.05 mmol/g). The charge is a contribution of dissociable carboxylic groups of CMC, which differ in their p*K*a values by 0.5 units (p*K*a_CNF_ = 3.5, p*K*a_CMC_ = 4.0), meaning that the CNF carboxylic groups are slightly more acidic. When translating this to the membranes consisting of only CNF and CMC in the absence of CA (7CNF/1CMC), one can observe that their surface charge value (0.67 mmol/g) was much closer to the one of neat CNF than the one of neat CMC, most probably due to disintegration of both components when exposed to wet conditions. This is in good agreement with the theoretical values of a 7 to 1 mixture of CNF and CMC, which amounted to 0.75 mmol/g. Most of the charges in this case were a contribution of the CNF carboxylic groups. Notably, the p*K*a value of 7CNF/1CMC (p*K*a_7CNF-1CMC_ = 4.8) was higher than the p*K*a of both single components, which can be consequence of the dehydration process during processing. In the case of the 4CNF/4CMC membrane without CA, the measurement was not possible due to complete disintegration.

On the other hand, for the 4CNF/4CMC membrane containing CA (1.07 mmol/g), one can observe an increase in surface charge when compared to 7CNF/1CMC (0.9 mmol/g). This is due to the higher amount of CMC in the membrane in the former case. The theoretical value of the 4 to 4 mixture of CNF and CMC amounted to 1.93 mmol/g, which is almost 2-fold higher than the experimental value. This is an indicator of successful cross-linking of CNF and CMC by citric acid, which results in a reduction in the number of dissociable carboxylic groups due to covalent bonding. 

**Figure 4:**
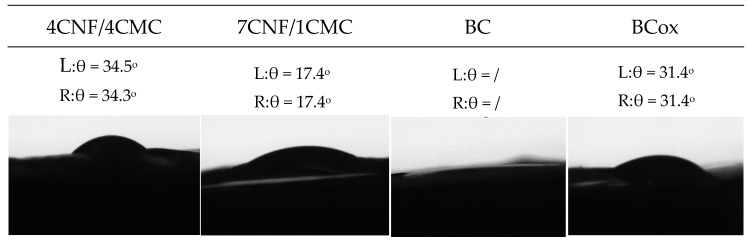
Contact angle (θ) and respective photographs of diverse membranes.

Contact angle measurement is a simple procedure for fast estimation of wettability, important in applications such as dye adsorption. The goniometry data with respective images on Figure 4 confirm the expected intrinsic hydrophilicity of all cellulose membranes, with the smallest contact angle measured in the BC membrane, where immediate drop spelling was depicted. In addition, the presence of CMC with a small substitution degree, carrying the partially hydrophobic CH_2_COO^−^ moiety, increased the angle 2-fold with a 4 times higher mass of CMC, at the expense of CNF. The oxidation process also significantly increased the contact angle due to the different nature of CHO and OH groups, as well as the crystallinity and crystal orientation, as surface interactions of different crystal faces of pure cellulose differed greatly with respect to the availability of OH groups. 

**Figure 5:**
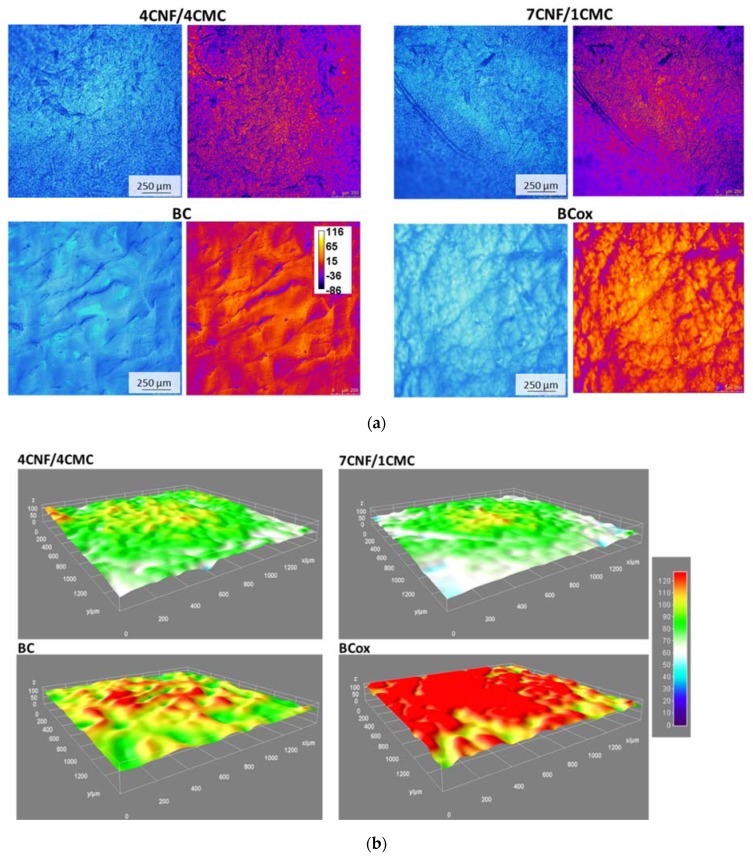
(**a**) CFM micrographs of cellulose membranes, designated as 4CNF/4CMC, 7CNF/1CMC, BC, and BCox, depicted in a fully swollen state with respective color-coded surface images from the fluorescent channel. The calibration bar (in µm) corresponds to different distances from the optical plane in focus. (**b**) 3D thermal surface plots with the calibration bar (in µm) on the right corresponding to different roughness levels.

The wettability of surfaces is largely dependent on the surface morphology and µ-roughness, which affects the drop size and shape, thus defining the ultimate angle. Membrane morphology in the wet state was visualized by CFM, utilizing the cellulose autofluorescence as presented in Figure 5a. Indeed, according to a recent study [37], the source of the nanocellulose and the chemical treatment guide its optical properties; therefore, its autofluorescence can exist at various (blue-to-yellow) wavelengths. For better visualisation of surface roughness, the color-coded images related to µ-roughness features are presented next to micrographs from the fluorescent signal. They reveal very distinct profiles among different membranes. Non-uniformly dispersed CNFs form large bundles in the case of a low CMC content (7/2), while a higher CMC presence (4/4) improved the CNF dispersibility, probably due to the extensive H-bonding and Van der Waals forces during the casting process. The membrane shrinking was most visible in BCox, as a consequence of membrane oxidation, especially when compared with much flatter BC. More detailed inspection of these qualitative findings was performed by the plotting of the 3D surface (Figure 5b) of the CFM image from the fluorescence channel using the ImageJ plugin Interactive 3D Surface Plot, which enables the representation of 3D topography, while the additional SurfaceJ plugin quantitatively mapped the surface topography by measuring the µ-roughness parameters [38]. The latter exhibited roughness (in µm) at max. peak (*Rp*), valley (*Rv*), and total (*Rt*). The color-coding visually identified the highest µ-roughness of 4CNF/4CMC as *Rt* = 179 µm, also with a high peak (*Rp* = 131 µm) and valley (*Rv* = −43 µm). The lowest values were measured for BC (99 µm, 39 µm, and −60 µm, for *Rt*, *Rp,* and *Rv*, respectively). This finding corresponds to the highest and lowest contact angle measured for 4CNF/4CMC and BC, respectively.

**Figure 6:**
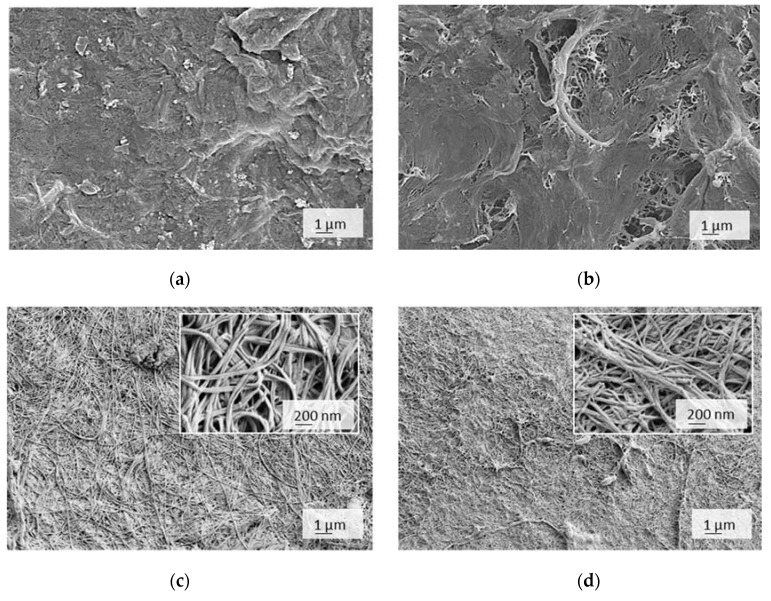
SEM micrographs of cellulose membranes, designated as (**a**) 4CNF/4CMC, (**b**) 7CNF/1CMC, (**c**) BC and (**d**) BCox, depicted in a dry state.

SEM imaging performed under a dry state delivered high-resolution micrographs presented in Figure 6. It can be identified that BC-based membranes largely differed from the CNF/CMC-based one, the latter being much more heterogeneous and less compact than finely nanostructured BC. 

The non-uniformly dispersed CNFs, forming large bundles and irregular pores at the bottom surface in the case of a low CMC content, are depicted, while higher CMC addition improved the CNF dispersibility whilst reducing the porosity due to the extensive H-bonding and Van der Waals forces. Both forces require molecular contact (OH group distance of 0.25–0.35 nm) [39], which occurs during the casting process. There was a typical BC network structure with unique micromorphology and isotopically and loosely arranged nanofibers, which were closely interconnected, but nanoporosity was evident, and it allowed the wetting at the first instance as well as a larger active surface for adsorption. The BC microstructure was not significantly compromised after the oxidation process; however, merging between neighbouring nanofibrils occurred, as observed within higher magnification inserts, which translated into a reduction in nanoporosity, compacting, and shrinking at the macroscale. As suggested by FTIR, this could have been a consequence of reduced crystallinity, allowing closer intermolecular contact and H–bonding between CHO and surrounding OH groups.

### 3.3. Adsorption Efficiency and Kinetics 

**Figure 7:**
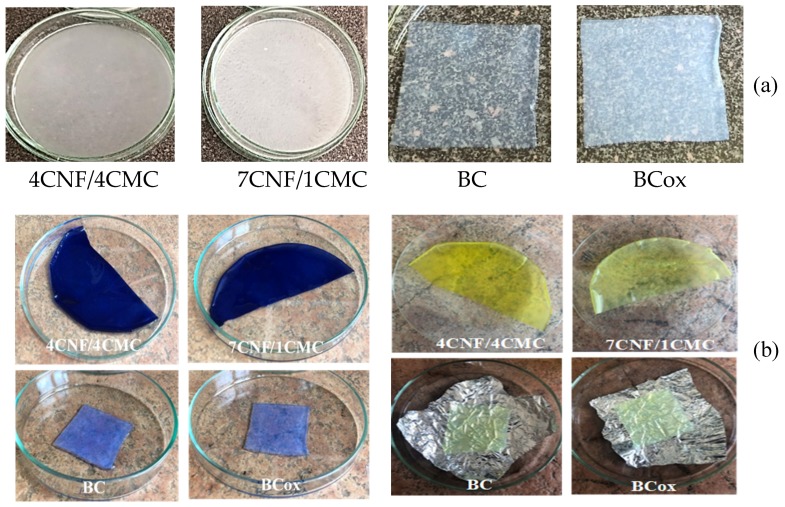

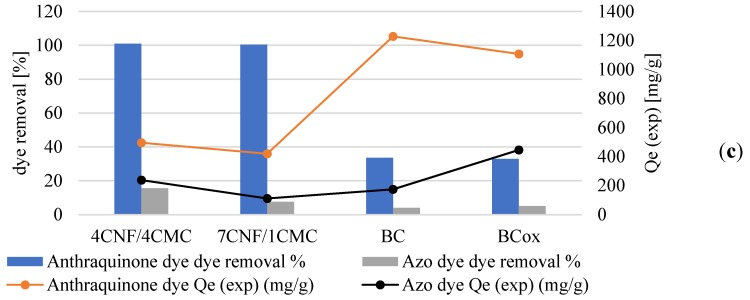
Cellulose membranes (**a**) before and (**b**) after immersion in anthraquinone (blue) and azo (yellow) dye solutions. (**c**) Mixed plot presenting dye removal (%) and equilibrated quantity of adsorbed dye Qe (mg/g) by different membranes.

The nature of the adsorption process depends on the physical or chemical characteristics of the adsorbent system and also on the system condition [40]. As can be initially visualised in Figure 7, the membrane adsorption was successful to different extents on different membranes, using different cationic dyes. Regarding the latter, much more intensive dye adsorption was observed in the case of anthraquinone (blue), compared to azo (yellow) dye, which was also confirmed by the results of the dye removal percentage (Figure 7c). Here, it is worth mentioning that dye removal analysis accounted for the normalised membrane surface (20 cm^2^), and not the dry adsorbent weight, and results revealed similar values between CNF/CMC with different ratios and BC–BCox in the case of anthraquinone dye, with the first two combinations being threefold more effective than the second type. This we assume to be a consequence of higher water binding to BC and BCox itself, which have a very high swelling degree (~1500% and ~1200%, respectively [25]), which is not the case with CNF/CMC, where adsorption was more preferable due to lower initial hydration. Another important reason was the much lower surface charge of BC and BCox compared to CNF/CMC membranes, presented in the titration data. In the case of azo dyes, the most effective was 4CNF/4CMC, where the effect of the highest surface charge was pronounced. The anthraquinone dye had a 30 g/mol higher *M*w than azo dye, which contributed to its substantivity towards the membrane surface. We assume that the difference in ionization constants of anthraquinone and azo dyes at the pH at which the adsorption experiment was undertaken (pH 5) is another reason for the very pronounced difference in the removal efficiency of the dyes. Azo dyes have one or more azo chromophores (N=N) and bonds between two or more aromatic rings. On the other hand, anthraquinone dyes have chromophore groups (=C=O and=C=C=), forming an anthraquinone complex. Looking at the exact molecular structure of both dyes (Figure 1), it can be assumed that azo dyes carry a positive charge, delocalized over the chromogen, while in the case of anthraquinone dyes, the non-protonated ammonium group on the one side and the quaternary ammonium group on another contribute to localized positive charge [41] under the acidic condition applied in the adsorption experiment. The charge localization is expected to provide stronger ionic interaction with the negative charge on cellulosic membranes, leading to a higher adsorption effect.

Comparing the *Qe* values, where the normalisation was done based on dry weight (mg dye per g adsorbent), the results were in favor of BC with the highest anthraquinone dye adsorption (1228 mg/g) but were lowest for 7CNF/1CMC (419.24 mg/g), while in the case of azo dye, the BCox was most effective, with 445.7 mg/g. It is obvious that membranes with the highest surface charge do not dominate in dye removal or in adsorption efficiency. This indicates the influence of other factors on the dye adsorption process, such as the microstructure of the membranes.

The adsorption efficiency depends on the contact time of the solid–liquid interface in the diffusion process. The relationship between the initial dye concentration and adsorption rate can be represented by adsorption kinetics, applied separately for anthraquinone and azo dyes at the same initial concentration (5 mg/L). Two simplified models, the pseudo-first order (presented by Equation (3)) and pseudo-second order (presented by Equation (4)), were used:

(3) $$\ln(q_{e}-q_{t})=lnq_{e(th)}-k_{1}t$$

(4) $$\frac{t}{q_{t}}=\frac{1}{k_{2q^{2}e(th)}}+\frac{t}{q_{e(th)}}$$

where *k*_1_ (min^−1^) and *k*_2_ (g/mg min) are the rate constants of both models, while *Qe*(*exp*) (mg/g) and *Qe*(*th*) (mg/min) are the amounts of the respective dye (mg) adsorbed per gram of adsorbent under experimental conditions and the theoretical value calculated from the model, respectively. 

**Figure 8:**
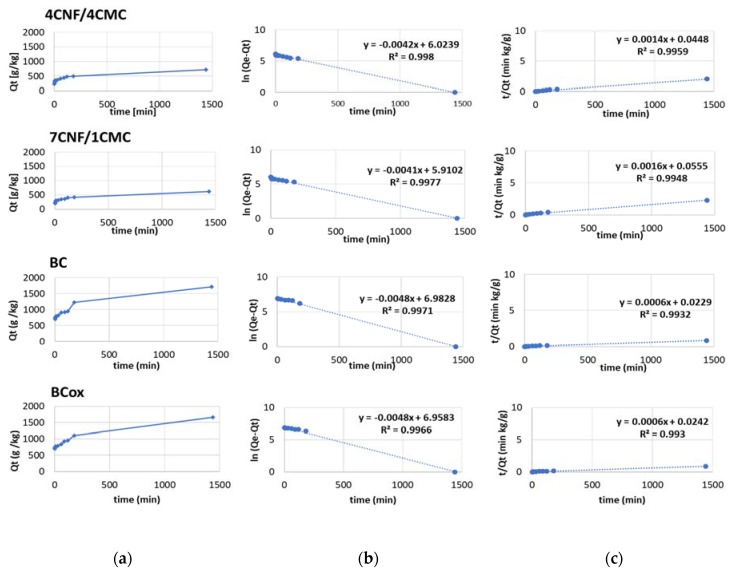
(**a**) Adsorption behavior of anthraquinone dye C.I. Basic Blue 47, (**b**) pseudo-first-order kinetic plots, and (**c**) pseudo-second-order kinetic plots of 4CNF/4CMC, 7CNF/1CMC, BC, and BCox membranes.

Comparing the values of linear regression coefficients *R*^2^ between both models for anthraquinone dye (Figure 8), higher values (in the range 0.96–0.99) were obtained for pseudo-second-order models, whereas the lowest values were obtained for BC and BCox for the pseudo-first-order model (0.6484 and 0.7131, respectively). Moreover, the values of experimentally obtained *Qe*(*exp*) were similar those of *Qe*(*th*) in the case of the pseudo-second order kinetic model, which confirms the applicability of the pseudo-second-order rate equation for the adsorption of anthraquinone dye C.I. Basic Blue 47 on all membranes. Based on this model and comparing the kinetic coefficient (*k*_1_) extracted from the curve slope, the highest value was obtained for the 7CNF/1CMC membrane, meaning the fastest adsorption kinetic rate in this case. The BC and BCox membranes had identical kinetic rates, lower than those of CNF/CMC membranes.

**Figure 9:**
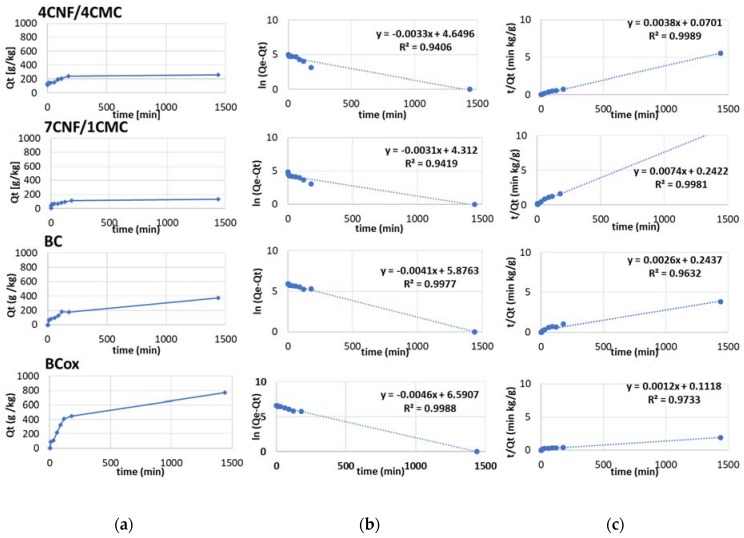
(**a**) Adsorption behavior of azo dye C.I. C.I. Basic Yellow 29, (**b**) pseudo-first-order kinetic plots, and (**c**) pseudo-second-order kinetic plots of 4CNF/4CMC, 7CNF/1CMC, BC, and BCox membranes.

Linear regression coefficients *R*^2^ between the models for azo dye (Figure 9) were slightly different; higher values (in the range 0.90–0.97) were obtained for pseudo second-order models for all membranes except the BCox where higher *R*^2^ (0.88) was obtained for the pseudo first-order model. This confirms the applicability of the pseudo second-order model for all membranes, excluding the BCox, where more complex adsorption processes can be assumed. Based on the kinetic coefficient (k1), the fastest adsorption kinetic rate obtained (the same as in the case of anthraquinone dye) was for the 7CNF/1CMC membrane.

## 4. Conclusions

Eco-friendly, fully cellulose-based membranes were prepared as highly efficient, cationic dye adsorbents, using two facile procedures, i.e., solvent-casting with citric acid-mediated esterification and periodate oxidation. Membranes with different surface charges, µ-roughness, and morphological features were obtained, all modulating the dye adsorption process in term of efficiency (Qe) and kinetic parameters. Excellent (~100%) dye removal efficiency for both CNF/CMC-based membranes was obtained, while BC and BCox adsorbed only 24.3% and 23.6%, respectively, when anthraquinone dye was used. Azo dye was only adsorbed with an efficiency of 7–9% on CMC/CNF-based membranes compared with 5.57% on BC and 7.33% on BCox membranes, implying specificity of membranes towards specific type of molecules. Applicability of a pseudo-second-order model was confirmed for both dyes and all membranes, except for BCox in combination with azo dye, demonstrating the fastest adsorption rate in the case of the 7CNF/1CMC membrane. Moreover, the adsorption efficiency at equilibrium in the case of BC (1228 mg/g) was an order of magnitude higher than that of reported bio-based [42] and non bio-based adsorbents, implying high potential of this cost-effective and eco-friendly material in the field of waste-water management. One focused application of membranes is their action as re-usable adsorption layers on top of available filtration membranes.
